# The KPZ Equation of Kinetic Interface Roughening: A Variational Perspective

**DOI:** 10.3390/e28010055

**Published:** 2025-12-31

**Authors:** Horacio S. Wio, Roberto R. Deza, Jorge A. Revelli, Rafael Gallego, Reinaldo García-García, Miguel A. Rodríguez

**Affiliations:** 1 Institute for Cross-Disciplinary Physics and Complex Systems (IFISC), UIB-CSIC, Universitat de les Illes Balears, E-07122 Palma de Mallorca, Spain; 2 IFIMAR, FCEyN-UNMdP and CONICET, Mar del Plata B7602AYL, Argentina; deza@mdp.edu.ar; 3 IFEG, FaMAF-UNC and CONICET, Cordoba X5000HUA, Argentina; jorge.revelli@unc.edu.ar; 4 Department of Mathematics, Gijon Campus, University of Oviedo, E-33203 Gijon, Spain; rgallego@uniovi.es; 5 Department of Physics and Applied Mathematics, School of Sciences, University of Navarra, E-31008 Pamplona, Spain; regarciag@unav.es; 6 Instituto de Física de Cantabria (IFCA), CSIC, University of Cantabria, E-39005 Santander, Spain; rodrigma@ifca.unican.es

**Keywords:** kinetic interface roughening, KPZ equation, variational approach

## Abstract

Interfaces of rather different natures—as, e.g., bacterial colony or forest fire boundaries, or semiconductor layers grown by different methods (MBE, sputtering, etc.)—are self-affine fractals, and feature scaling with *universal* exponents (depending on the substrate’s dimensionality *d* and global topology, as well as on the driving randomness’ spatial and temporal correlations but *not* on the underlying mechanisms). Adding lateral growth as an essential (non-equilibrium) ingredient to the known equilibrium ones (randomness and interface relaxation), the Kardar–Parisi–Zhang (KPZ) equation succeeded in finding (via the dynamic renormalization group) the correct exponents for flat d=1 substrates and (spatially and temporally) uncorrelated randomness. It is this *interplay* which gives rise to the unique, non-Gaussian scaling properties characteristic of the specific, universal type of non-equilibrium roughening. Later on, the asymptotic statistics of process h(x) fluctuations in the scaling regime was also analytically found for d=1 substrates. For d>1 substrates, however, one has to rely on numerical simulations. Here we review a variational approach that allows for analytical progress regardless of substrate dimensionality. After reviewing our previous numerical results in d=1, 2, and 3 on the time evolution of one of the functionals—which we call the *non-equilibrium potential* (NEP)—as well as its scaling behavior with the nonlinearity parameter λ, we discuss the stochastic thermodynamics of the roughening process and the memory of process h(x) in KPZ and in the related Golubović–Bruinsma (GB) model, providing numerical evidence for the significant dependence on initial conditions of the NEP’s asymptotic behavior in both models. Finally, we highlight some open questions.

## 1. Introduction

Originally formulated in 1986 to describe kinetic interface roughening, the nonlinear stochastic partial differential equation known as the Kardar–Parisi–Zhang (KPZ) equation readily became a paradigm of non-equilibrium dynamic critical scaling systems, defining as its canonical member the rather comprehensive KPZ universality class. This foundational work [1] established a profound connection between the dynamics of interface growth and the broader field of non-equilibrium physics. The mathematical formulation of the KPZ equation initially posed significant challenges, as its strong nonlinearity exceeded the scope of existing methods for studying stochastic partial differential equations. A major breakthrough in addressing the “well-definedness” of this equation occurred in 2013 [2]. The resolution of this fundamental mathematical problem was essential for advancing the analytical understanding of complex stochastic systems and extracting deeper physical insights.

Universality is a cornerstone concept in statistical physics, asserting that diverse physical systems and mathematical models can exhibit identical statistical behaviors in their long-time and large-scale limits, irrespective of their specific microscopic details. The KPZ equation describes and connects a broad spectrum of significant stochastic growth-like processes across physics, chemistry, and biology, spanning both classical and quantum systems. This wide applicability means that the KPZ universality class offers a powerful coarse-grained predictive framework, enabling the inference of macroscopic behaviors across diverse systems without requiring detailed knowledge of their microscopic interactions. Researchers can infer macroscopic behaviors and statistical properties across vastly different physical, chemical, and biological systems without needing to delve into their specific microscopic interactions. This “surprising and broad universality” of the one-dimensional KPZ equation (even appearing in deterministic Hamiltonian systems) further elevates its status from a mere model to a fundamental principle that unifies the study of non-equilibrium phenomena, suggesting that common mathematical structures dictate emergent behavior irrespective of the underlying physical substrate.

The study of the KPZ equation has revealed deep and unexpected mathematical connections that have been pivotal in its analytical understanding and in broadening its perceived universality, due to its relation with both the Burgers equation of turbulence and the restricted partition function of directed polymers in random media (DPRM). In particular, the KPZ equation and its associated universality class have remarkably extended their reach into the domain of biological growth phenomena, providing a framework for understanding complex pattern formation and spatial dynamics in living systems. The KPZ equation is being increasingly applied to movement ecology due to its ability to model complex stochastic processes that resemble ecological patterns. Since the scaling behavior of the surface of *Escherichia coli* and *Bacillus subtilis* colonies growing on agar plates was established to be self-affine and consistent with models in the so-called “KPZ universality class” [3], there have been numerous applications of the KPZ equation in movement ecology [4,5], among which we might cite the following:

*Modeling nonlinearities in ecological systems.* The KPZ equation’s inclusion of nonlinear growth terms is particularly useful for modeling ecological processes where interactions between individuals or between species and their environments lead to complex, emergent movement patterns. For example, the propagation of invasive species, herd dynamics, or predator–prey interactions often exhibit such nonlinearities, and KPZ can capture these effects efficiently. A foundational exploration of movement patterns, emphasizing the need for nonlinear models like the KPZ to understand large-scale spatial patterns, can be found in [6].
*Spatial pattern formation in ecosystems.* KPZ-like models can explain spatial clustering and aggregation, phenomena common in ecosystems due to habitat fragmentation or social behaviors in species. In this sense, the KPZ equation aligns well with empirical observations in movement ecology, where spatially heterogeneous environments cause uneven dispersal patterns. Book [7] elaborates on spatial pattern formation and how stochastic models, including those similar to KPZ, apply to understanding the distribution and aggregation of organisms.
*Capturing anomalous diffusion in animal movement.* Traditional diffusion models often fail to capture the complexity of animal movement, which frequently exhibits subdiffusive or superdiffusive behavior due to factors like resource hotspots or behavioral adaptations. The KPZ equation, with its growth and roughening terms, provides a more flexible framework for modeling these deviations from classical diffusion. The article [8] reviews the inadequacy of simple diffusion models and how alternative stochastic approaches like KPZ are better suited to describe animal search behaviors that diverge from Brownian assumptions.
*Modeling stochastic processes in animal movement.* The KPZ equation is an excellent tool for capturing random yet structured movement patterns, such as those seen in animal migrations or dispersal behaviors. Unlike simple random walks, the KPZ framework can describe anisotropic movement, a key aspect of ecological processes where environmental and physiological conditions lead to non-uniform patterns. The article [9] discusses the role of stochastic models, like KPZ, in modeling animal movement patterns beyond simple Brownian motion.
*Interfacing with statistical mechanics for predictive ecology.* KPZ’s roots in statistical mechanics make it a natural fit for ecological models that require predictions based on system dynamics under uncertainty. By using KPZ, researchers can leverage known statistical properties to predict critical transitions, tipping points, or responses to environmental changes. The article [10] discusses the interface between movement ecology and statistical mechanics, highlighting how frameworks like KPZ facilitate a predictive approach to ecological modeling.
In sum, the KPZ equation offers a robust framework for addressing the complex, nonlinear, and stochastic characteristics of movement patterns in ecology. Its applicability extends beyond traditional diffusion models, making it valuable for studying the spatial and temporal heterogeneity observed in animal populations. By leveraging the KPZ equation, ecologists can create models that more accurately reflect real-world movement dynamics, supporting better predictive and management tools in ecological research [11,12,13,14]. The KPZ universality class is characterized by definite values of *either* the *roughness exponent* *α or* the *dynamical exponent z*, *and* by the relation α+z=2. If *L* denotes the substrate’s *length* along *any* dimension, then the global width
  $$W\left(L,t\right):={\langle \left\{{\left[h\left(x,t\right)-\left\{h\right\}\left(t\right)\right]}^{2}\right\}\rangle }^{1/2}$$
  scales as $L^{\alpha }$. Here *h* is the local height (flat substrates) or radius (curved substrates), and {…} denote spatial average and 〈…〉 the ensemble one. Moreover, the correlation length ξ(t) on the interface scales as $t^{1/z}$. For finite *L*, the scaling regime ends at *saturation*, when ξ∼L. As L→∞, it is a dynamical critical phenomenon. Putting it all together, it turns out that in the scaling regime $W\left(L,t\right)∼t^{\beta }$, where β:=α/z is called the *growth exponent*.

Several kinetic models (such as the Eden model for bacterial colonies) yielded values consistent with the set α=1/2, z=3/2 (hence β=1/3) for flat 1d substrates. The two necessary ingredients can be abstracted from the kinetic models: *driving environmental fluctuations* and *interface relaxation*. Note that the requirement of space- and time-continuous models to respect basic symmetries (*t*-, x-, and *h*-translations plus x-rotation and inversion) leaves us only with terms of the form $\nabla^{2i}h$ (i.e., *i*-laplacians) or at most ${\left(\nabla h\right)}^{2i}$ if the equilibrium condition is relaxed. Among them, $\nabla^{2}h$ and ${\left(\nabla h\right)}^{2}$ are the most relevant operators in the sense of the renormalization group. In 1982, Edwards and Wilkinson (EW) [15] proposed (for granular aggregates on arbitrary-dimensional flat substrates) the following extended Langevin equation:

(1) $$\frac{\partial h(x,t)}{\partial t}=\nu \;\nabla^{2}h\left(x,t\right)+F+\xi \left(x,t\right),$$

as the continuum limit of those kinetic models. Here ν is a diffusion coefficient accounting for surface relaxation, and *F* is a particle flux. The stochastic field ξ(x,t), with 〈ξ(x,t)〉=0∀x,t and

$$\langle \xi \left(x,t\right)\xi \left(x^{\prime },t^{\prime }\right)\rangle =\varepsilon \delta \left(x-x^{\prime }\right)\delta \left(t-t^{\prime }\right),$$

is spatially uncorrelated Gaussian white noise accounting for driving environmental fluctuations. However, something was still missing since in 1d, the EW model yielded the correct value of α but not that of β. Moreover for F=0, the system remained in equilibrium.

Scarcely four years after, the missing relevant factor was identified as *lateral growth*, due to the fact that particles aggregate *normally* to the surface [1]. (The new term, gauged by parameter λ, is the lowest order in the expansion of the curvature.) This makes the linear stochastic partial differential Equation (1) into a nonlinear one, *the Kardar–Parisi–Zhang (KPZ) equation*.

(2) $$\frac{\partial h(x,t)}{\partial t}=\nu \;\nabla^{2}h\left(x,t\right)+\frac{\lambda }{2}{\left[\nabla h\left(x,t\right)\right]}^{2}+F+\xi \left(x,t\right)$$

(For readers less familiar with the KPZ equation, we note that it describes the temporal evolution of an interface h(x,t) under the combined effects of surface relaxation $\nu \nabla^{2}h$, nonlinear lateral growth $\frac{\lambda }{2}{\left(\nabla h\right)}^{2}$, and stochastic noise ξ(x,t). The richness of the emergent collective behavior from this simple yet non-trivial equation makes it a paradigm of non-equilibrium systems). Now for F=0, the system remains *out* of equilibrium and the interface moves at a rate depending on λ. Once they recognized that Equation (2) can be assimilated to a Burgers equation for ∇h(x,t), they realized that λ must remain invariant under dynamic renormalization, which led to the aforementioned relation α+z=2. Fortunately, for flat 1d substrates, the dynamic renormalization group fixed point (DRGFP) can be reached perturbatively. This allowed them to obtain analytically the aforementioned exponents α=1/2, z=3/2, β=1/3.

Of course, $\nabla^{4}h$ becomes the most relevant operator for ν=0. Golubović and Bruinsma [16] studied the effect of this on the lower critical dimension and the exponent α, and concluded that it leads to a different universality class than the KPZ one, showing that the ${\left(\nabla h\right)}^{2}$ term *does not suffice* (either by itself or through its interplay with the noise) to determine the universality class, but it is *their interplay* with interface relaxation that does.

Keeping two terms for interface relaxation (i.e., taking curvature effects into account), the growth rate equation reads

(3) $$\frac{\partial h(x,t)}{\partial t}=\nu \nabla^{2}h\left(x,t\right)-K\nabla^{4}h\left(x,t\right)+\frac{\lambda }{2}{\left(\nabla h(x,t)\right)}^{2}+\xi \left(x,t\right).$$

In Section 2, we review a variational formulation of the KPZ equation and related ones and point out some issues like the necessity of Galilean invariance in discrete versions, the Markov character of process h(x), and the existence of steady-state distribution (before reaching saturation!) in d=1 substrates. In Section 3, as a prolegomenon to Section 4, we recall previous numerical results on the time evolution of the NEP in d=1, 2, and 3, as well as its scaling behavior with the nonlinearity parameter λ. In Section 4 (always from a variational perspective), we show the intimate connection between the NEP and entropy production (both from a path-integral formulation or starting directly from the KPZ equation) and address the memory of process h(x) with respect to initial conditions, both in KPZ and GB. In Section 5, we highlight some open questions that can be approached from a variational perspective.

## 2. The Variational Formulation

### 2.1. Model A—Like Variational Formulation

Although describing a non-equilibrium process, Equation (2) originated in purely kinetic models. For some time, it was believed that its drift $\nu \;\nabla^{2}h\left(x,t\right)+\frac{\lambda }{2}{\left[\nabla h\left(x,t\right)\right]}^{2}$ cannot be derived from an effective free energy [17].

Using the Hopf–Cole transformation

$$\Psi \left(x,t\right):=\exp\left[\frac{\lambda }{2\nu }h\left(x,t\right)\right]$$

(mapping *h* onto a field Ψ obeying a linear equation, but with multiplicative noise), it was proved in [18] that *for any d*, Equation (2) can be cast as *model A* (namely, a relaxational system without conservation laws, in the nomenclature of [19])

(4) $$\frac{\partial h(x,t)}{\partial t}=-\Gamma \left[h\right]\frac{\delta F[h]}{\delta h(x,t)}+\xi \left(x,t\right),$$

with functional F[h] given by Equation (5), and functional Γ[h] (known as the *motility*) by

$$\Gamma \left[h\right]:={\left(\frac{2\nu }{\lambda }\right)}^{2}\exp\left[-\frac{\lambda }{\nu }h\left(x,t\right)\right].$$

(5) $$F\left[h\right]:=\int dx\exp\left[\frac{\lambda }{\nu }h\left(x,t\right)\right]\frac{\lambda }{2\nu }\left\{-Fh\left(x,t\right)+\frac{\lambda }{4}{\left[\nabla h(x,t)\right]}^{2}\right\}$$

**Figure 1:**
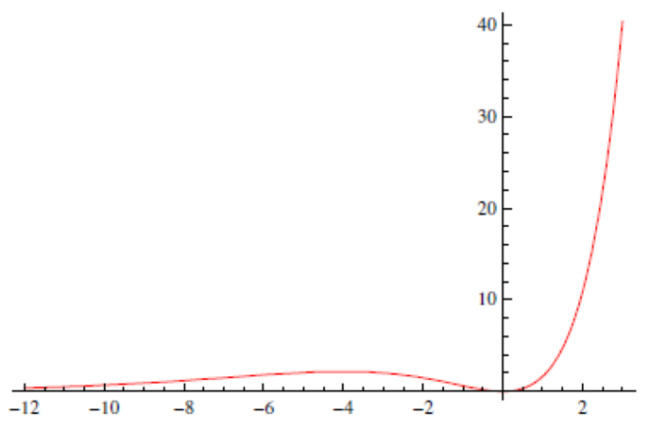
The effective potential f(h) of functional F[h] features a minimum at h=0 [with f(h)=0 for F=0]. The system evolves by overcoming the barrier at h<0 and continuing towards h→−∞, where again f(h)=0.

The Lyapunov property $\dot{F}\left[h\right]\leq 0$ is easy to check, and the minimum of F[h] is reached by constant functions. In addition, the *effective potential* f(h)—the restriction of F to h(x,t)=const.—is minimum at h=0 for F=0 (Figure 1). Hence, F[h] is a *Lyapunov functional* for the KPZ problem, and the deterministic KPZ equation displays simple dynamics: the asymptotic stability of constant solutions indicates that arbitrary initial conditions approach constant profiles for long times. While the deterministic case is simple, the stochastic dynamics remain highly non-trivial, producing self-affine fractal profiles. Notably, the Lyapunov functional offers little intuition about these stochastic features.

As shown in [18], F[h] and Γ[h] (together with the density $\tilde{F}\left[h,\nabla h\right]$, defined by $F\left[h\right]:=\int \tilde{F}\left[h,\nabla h\right]dx$) fulfill the relations postulated in [20] on the grounds of global shift invariance.

It is worth noting that several studies [21,22,23,24] have shown that in oscillator systems subjected to noise, synchronization is achieved when the coupling is strong enough. This dynamic process can be related to a roughness process and exhibit, in one dimension, scale invariance within the KPZ universality class. Since Kuramoto-type models can be obtained from energy functionals, this provides further support for the idea of the existence of such a functional for KPZ.

### 2.2. Discrete Version

Discretization is a necessity if the substrate dimensionality is d>1, where the DRGFP is not reachable perturbatively and one must resort to numerical integration to find the critical exponents. In the past, a criterion to judge the quality of a discretization of Equation (2) was that it should preserve the two main symmetries of Equation (2): *Galilean invariance* (GI) and a *fluctuation–dissipation relation* peculiar of 1d (1dFDR). Note that although there is an explicit FDR for diffusion phenomena, there is in principle no such thing for growth processes. Recently, however, a kind of FDR—similar to the diffusive one—was found for growth phenomena [25,26]. In turn, Galilean invariance implies that the physics of interface growth remains the same when viewed from a reference frame moving laterally at constant speed, provided the surface height is appropriately tilted. This symmetry protects parameter λ from being renormalized and imposes the constraint α+z=2.

**Figure 2:**
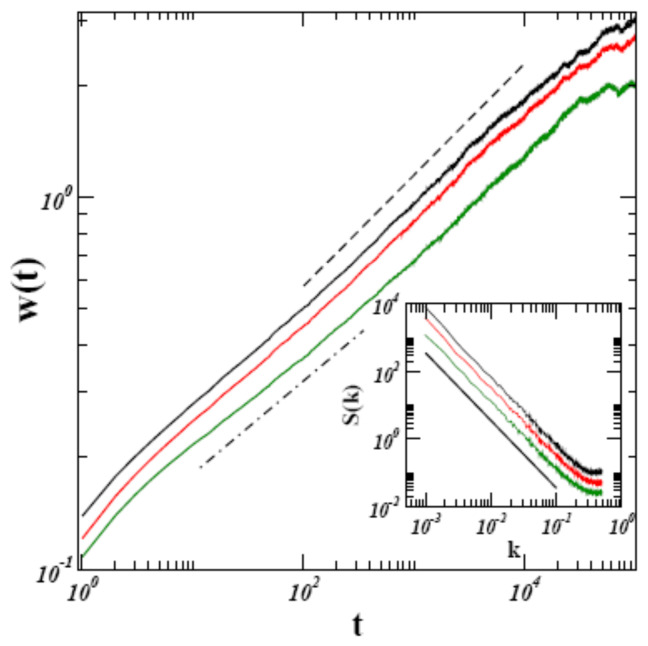
Numerical results for the global width (main graph) and the structure factor (inset) for λ=4, obtained with the alternative discretization scheme of [28] in a system of size L=1024, by averaging over 100 runs. Both results are consistent with the KPZ scaling, as indicated by the eye-guiding dashed line (β=0.3) in the main graph and the black solid line in the inset [−(2α+1)=2].

Consistency in employing the Hopf–Cole transformation constraints the discrete version of ${\left(\nabla h\right)}^{2}$ to have the form $\frac{1}{2}\left[{\left(h_{i+1}-h_{i}\right)}^{2}+{\left(h_{i-1}-h_{i}\right)}^{2}\right]$. In [27], scaling was obtained to a very good approximation (Figure 2) using this discretization, which breaks GI and 1dFDR explicitly.

### 2.3. Memory of Process h(x,t)

From the Family–Vicsék scaling of roughening processes [29], it turns out that

(6) $$h\left(x,t\right)∼v_{\infty }t+At^{\beta }\chi \left(x^{\prime }\right),\;x^{\prime }=B\;\frac{x}{t^{1/z}}.$$

Over the last 15 years or so, numerous works by mathematicians and physicists [2,30,31,32,33,34,35] have shown that the asymptotic (in the scaling regime) probability distribution P(χ) (for any *x*) not only has skewness, but shows *a remarkable dependence on the initial conditions*. In other words, process h(x) has memory.

Equation (2) is an extended Langevin equation; thus, the *joint process* [h](t) obeys a Fokker–Planck equation and is Markovian. Now h(x) obeys a marginal distribution and is *generically non-Markovian* (unless a conservation law, like, e.g., $\int dx\nabla^{2}h=0$ in the EW model, forces it to be Markovian).

Besides the *model A* variational formulation of Equations (4) and (5), Equation (2) can also be cast (again, *for any d*) as [18]

(7) $$\frac{\partial h(x,t)}{\partial t}=-\frac{\delta \Phi [h]}{\delta h(x,t)}+\xi \left(x,t\right),$$

with

(8) $$\Phi \left[h\right]:=\frac{\nu }{2}\int dx{\left(\nabla h\right)}^{2}-F\int dx\;h\left(x,t\right)-\frac{\lambda }{2}\int dx\int_{h_{\mathrm{ref}}}^{h(x,t)}D\psi {\left(\nabla \psi \right)}^{2}.$$

**Figure 3:**
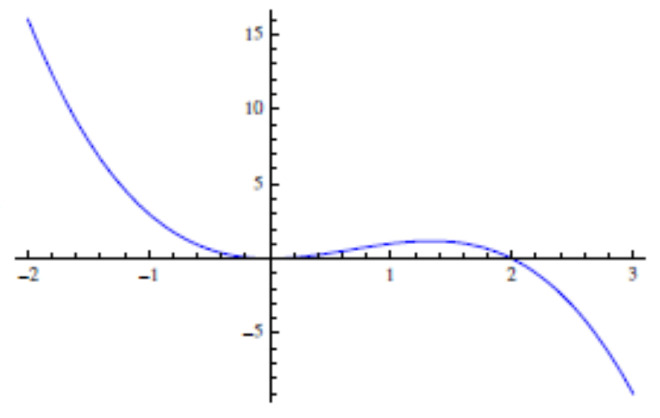
The effective potential ϕ(h) of Φ[h] also features a minimum at h=0 [with ϕ(h)=0 for F=0], but it is unbounded from below. The system evolves by overcoming the barrier at h>0 and rolling down to ϕ(h)→−∞ as h→−∞.

Again, $\dot{\Phi }\left[h\right]\leq 0$ and its effective potential is minimum at h=0 for F=0 (Figure 3). But at variance with F[h], Φ[h] is not bounded from below, so it is *not* a Lyapunov functional for h(x,t). However, Φ[h] *does* provides intuition on the stochastic dynamics (for instance, it resembles an extended escape problem).

In particular, Equation (8) can be transformed into

(9) $$\Phi \left[h\right]=\frac{\nu }{2}\int dx{\left(\nabla h\right)}^{2}-F\int dx\;h\left(x,t\right)-\frac{\lambda }{2}\int dx\int_{0}^{t}ds\left[\partial_{s}h\left(x,s\right)\right]{\left[\nabla h(x,s)\right]}^{2}.$$

which shows explicitly that Φ[h] *depends on the whole trajectory* [h](s), from 0 to *t*.

As in the case of reaction–diffusion systems, Equation (2) looks *formally* as a stochastically forced gradient flow (in the sense that the coupling terms have the same form for all sites), but it is not strictly gradient. Equation (7) is regarded as a gradient flow in the sense that its motility is Γ[h]=1.

### 2.4. On the Existence of a Stationary State for Process h(x,t)

The EW model of Equation (1) is an equilibrium system and as such, its joint probability distribution functional (pdf’l) P[h](t) is granted a stationary state $P^{\mathrm{stat}}\left[h\right]$. According to conservation law, $\int dx\nabla^{2}h=0$, P(h) is also stationary for any *x*. In the KPZ model, P[h](t) also obeys a Fokker–Planck equation, but since its collective mode grows steadily in the scaling regime [see Equation (6)], process [h](t) cannot be stationary in that regime—what is stationary is P(χ), as already argued. In principle, there is no theoretical reason why—even in 1d and with PBC—Equation (2) should attain a steady state $P^{\mathrm{stat}}\left[h\right]$. Yet less that $P^{\mathrm{stat}}\left[h\right]$ be the same as that of the (equilibrium) EW model. (Moreover, recall that most results indicating *strong dependence on initial conditions* have been obtained for 1d substrates.)

It is just a fact that, by imposing $\partial_{t}P\left[h\right]\left(t\right)=0$ in the functional Fokker–Planck equation for P[h(x),t|0,0]

(10) $$\frac{\partial P[h(x,t)]}{\partial t}=\int dx\frac{\delta J[h]}{\delta h},\;J\left[h\right]:=\frac{\delta \Phi [h]}{\delta h}\;P\left[h\left(x,t\right)\right]+\varepsilon \;\frac{\delta P[h(x,t)]}{\delta h},$$

it admits as a stationary solution for 1d substrates and periodic boundary conditions (PBCs) the exponential $\exp\{-\Phi_{EW}\left[h\right]/\varepsilon \}$ of the EW Ginzburg–Landau functional

$$\Phi_{EW}\left[h\right]:=\frac{\nu }{2}\int_{\Omega }dr\;{\left(\nabla h\left(r,\tau \right)\right)}^{2}.$$

This is what is meant by the abovementioned 1dFDR [17].

The novelty is that Equation (10) *also* admits as a stationary solution (for *any* substrate dimensionality and independently of BC) the exponential

$$P_{KPZ}^{\mathrm{stat}}\left[h\right]\propto \exp\left\{-\Phi_{KPZ}\left[h\right]/\varepsilon \right\},$$

with $\Phi_{KPZ}\left[h\right]$ given by Equation (8). Hence $\Phi_{KPZ}\left[h\right]$ is by definition a *non-equilibrium potential* (NEP) [36]. Clearly, this is a *formal* solution since $P_{KPZ}^{\mathrm{stat}}\left[h\right]$ is *not* normalizable, but $\exp\{-\Phi_{EW}\left[h\right]/\varepsilon \}$ is also formal. In fact, using the *short-time propagator* $P[h_{f},t_{i}+\tau |h_{i},t_{i}]$ obtained in a path-integral representation, namely

(11) $$\begin{array}{ccc} & & \ln P\left[h_{f},t_{i}+\tau |h_{i},t_{i}\right]\approx -\frac{\tau }{4\varepsilon }\int dx\left\{\frac{\partial h}{\partial t}+\frac{\delta \Phi [h]}{\delta h}\right\}\\ & & \approx -\frac{1}{4\varepsilon \tau }\int dx{\left(h_{f}-h_{i}\right)}^{2}-\frac{1}{\varepsilon }\left(\Phi \left[h_{f}\right]-\Phi \left[h_{i}\right]\right)-\frac{\tau }{4\varepsilon }\int dx\left[{\left(\frac{\delta \Phi_{f}}{\delta h}\right)}^{2}-{\left(\frac{\delta \Phi_{i}}{\delta h}\right)}^{2}\right], \end{array}$$

it becomes clear that

$$\int Dh_{i}\;P^{\mathrm{stat}}\left[h_{i}\right]\;P\left[h_{f},t_{i}+\tau |h_{i},t_{i}\right]$$

does not coincide with $P^{\mathrm{stat}}\left[h_{f}\right]$, indicating that $\exp\{-\Phi_{EW}\left[h\right]/\varepsilon \}$ is at most *marginally* stable.

## 3. KPZ NEP’s Time Behavior and Its λ-Dependence

We recall our numerical results on the time behavior of the NEP in dimensions d=1,2,3 [37]. These results (i) provide the first systematic characterization of the dimensional dependence of NEP, (ii) quantify memory effects in NEP that had not been reported, and (iii) establish explicit connections with stochastic thermodynamics.

**Figure 4:**
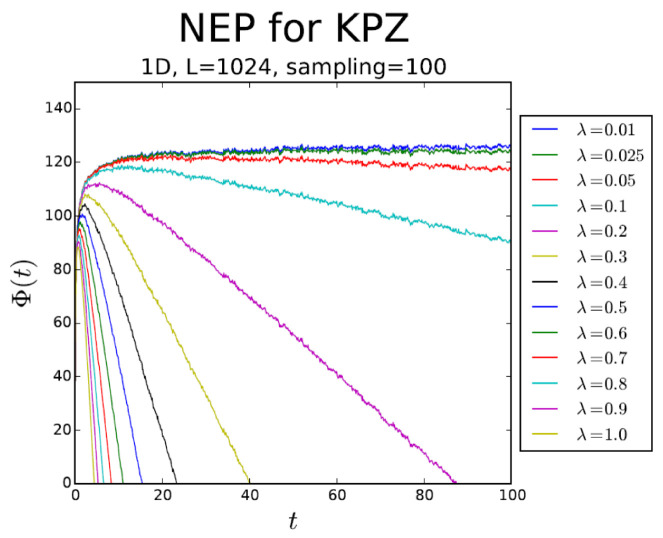
$\bar{\Phi }$ vs. *t* in 1d. The (absolute value of the) slope of the asymptotic behavior increases as $\lambda^{2}$.

**Figure 5:**
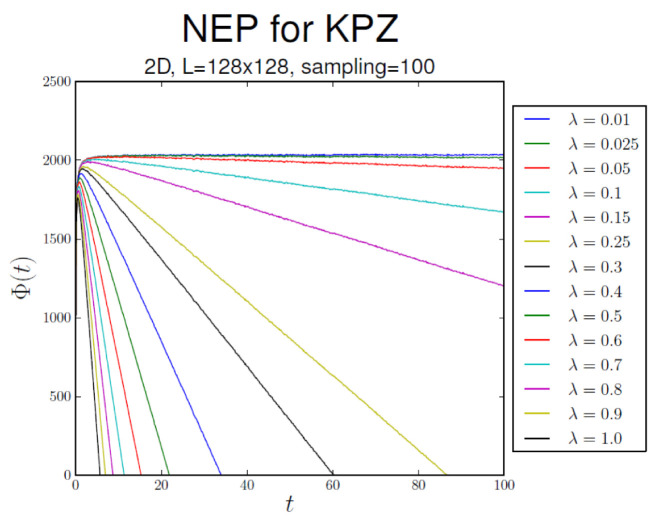
$\bar{\Phi }$ vs. *t* in 2d.

**Figure 6:**
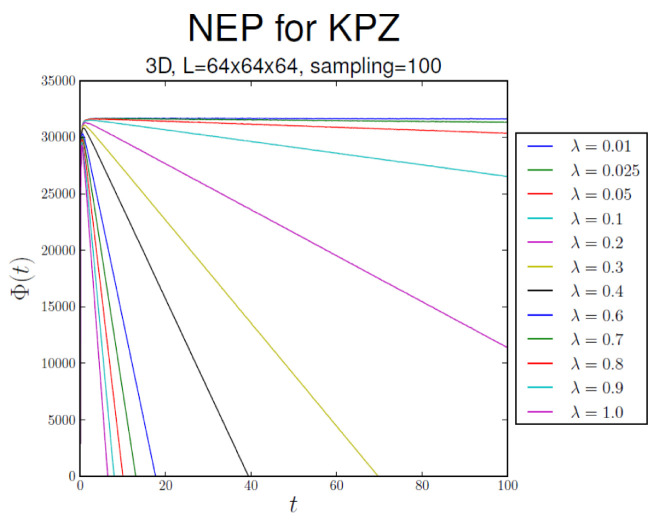
$\bar{\Phi }$ vs. *t* in 3d.

Figure 4, Figure 5 and Figure 6 display the behavior of

$$\bar{\Phi }\left(t\right):=\langle \Phi \left[h\left(x,t\right)\right]\rangle ,$$

that is, the average of Φ[h(x,t)] over realizations of process h(x,t), in substrate dimensionalities d=1, 2, and 3, respectively. In all cases, $\bar{\Phi }\left(t\right)\propto -\lambda^{2}t$ asymptotically. The NEP also serves as a criterion for the EW–KPZ crossover, which can be estimated from the maximum of the curves for λ≠0.

**Figure 7:**
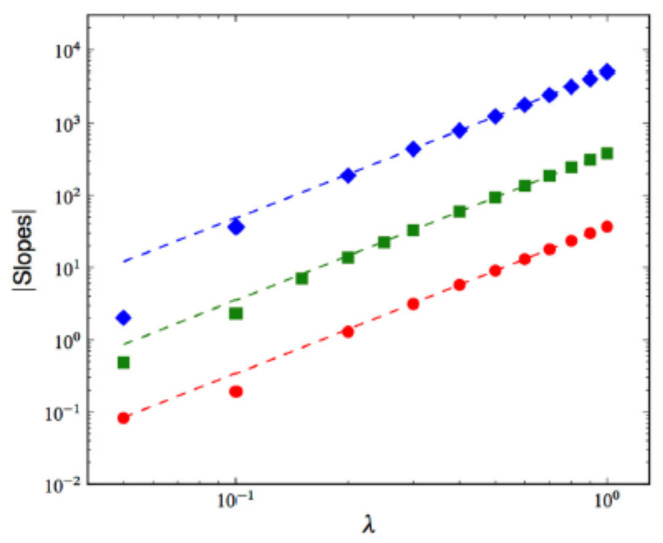
Mean λ-dependence over 100 samples of the NEP’s asymptotic slope. The symbols denote the substrate’s dimensionality: ⋄ 1d (size 1024), □ 2d (size $128^{2}$), and • 3d (size $64^{3}$). The dashed lines are best fits with $a\lambda^{b}$. b=2.01 ± 0.01∀d.

**Figure 8:**
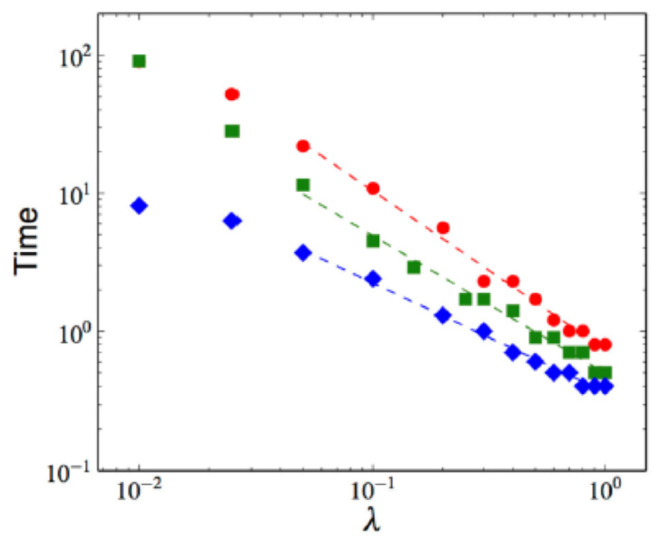
Mean λ-dependence over 100 samples of the time of occurrence of the NEP’s maximum, in • 1d (size 1024), □ 2d (size $128^{2}$), and ⋄ 3d (size $64^{3}$). Dashed lines are best fits with $a\lambda^{b}$ (b=1.14 in 1d, 0.98 in 2d, and 0.79 in 3d.).

Figure 7 and Figure 8 display the mean λ-dependence over 100 samples of the NEP’s asymptotic slope and of the time of occurrence of the NEP’s maximum, respectively, for d=1, 2, and 3. Whereas the first magnitude shows no dependence whatsoever on *d*, a mild d-dependence is seen in the second one.

We feel that, while RG fails for d>1, the variational approach could offer a qualitative or complementary insight for obtaining the critical exponents.

## 4. Novel Results

### 4.1. Stochastic Thermodynamics

From a path-integral approach [38,39], we proceed as follows: The probability distribution functional (pdf) of *a given* history (involving *h* at all sites) is

$$\begin{array}{ccc} & & P^{F}\left(\left[h\left(t\right)\right]|\left[h\left(0\right)\right]\right)=N^{F}\exp\left\{-S^{F}\left[h\right]\right\},\\ & & S^{F}\left[h\right]=\int_{0}^{t}ds\;L^{F}\left(\left[h\left(s\right)\right],\left[\partial_{s}h\left(s\right)\right]\right), \end{array}$$

where superscript *F* indicates increasing time sequence, $N^{F}$ is a normalization factor, and $S^{F}\left[h\right]$ is the *forward stochastic action* functional. The *forward stochastic Lagrangian*, given by Equation (12)

(12) $$L^{F}\left(\left[h\left(s\right)\right],\left[\partial_{s}h\left(s\right)\right]\right):=\frac{1}{4\varepsilon }\int_{\Omega }dx{\left(\partial_{s}h+\frac{\delta \Phi }{\delta h}\right)}^{2}$$

is still a functional of [h(t)]. We use calligraphic types for functionals of the configuration alone, and blackboard types for functionals of the whole history. The conditional pdf of tracing *the same* history *back* is

$$\begin{array}{ccc} & & P^{B}\left(\left[h\left(0\right)\right]|\left[h\left(t\right)\right]\right)=N^{B}\exp\left\{-S^{B}\left[h\right]\right\},\\ & & S^{B}\left[h\right]=\int_{0}^{t}ds\;L^{B}\left(\left[h\left(s\right)\right],\left[\partial_{s}h\left(s\right)\right]\right), \end{array}$$

with the *backward stochastic Lagrangian* given by Equation (13).

(13) $$L^{B}\left(\left[h\left(s\right)\right],\left[\partial_{s}h\left(s\right)\right]\right)=\frac{1}{4\varepsilon }\int_{\Omega }dx{\left(-\partial_{s}h+\frac{\delta \Phi }{\delta h}\right)}^{2}.$$

With the entropy production rate being $S^{F}\left[h\right]-S^{B}\left[h\right]$, one can readily obtain integral fluctuation theorems and thermodynamic uncertainty relations (TURs) [40,41].

Whereas the foregoing approach gives us a hint of the relevance of the NEP Φ[h] to the entropy production rate, the following approach starting directly from the KPZ equation enhances this relevance: We start expressing the KPZ equation in a semi-rigorous fashion (i.e., as one would write it in Itô interpretation if the noise was multiplicative), but explicitly taking into account that the KPZ equation is ill-posed when not regularized [2,42].

(14) $$dh\left(x,t\right)=\left[\nu \partial_{x}^{2}h\left(x,t\right)+\frac{\lambda }{2}{\left[\partial_{x}h\left(x,t\right)\right]}^{2}\right]dt+\sqrt{2D}\;dB\left(x,dt\right).$$

Here dB(x,dt) is the increment of a functional Wiener process, 〈dB(x,dt)〉=0, $\langle dB\left(x,dt\right)dB\left(x^{\prime },dt\right)\rangle =K_{l}\left(x-x^{\prime }\right)dt$, and we assume that the KPZ noise is spatially correlated over a small regularization length-scale *ℓ*. Although such regularization is usually omitted when introducing the KPZ equation, it appears in explicit calculations (in connection with a UV cut-off, e.g., a lattice).

For a KPZ equation with such a noise, the stochastic entropy change of the medium associated to a particular realization of the evolution of the KPZ front is given as [43]

(15) $$\Delta S_{t}^{m}\left[h\right]=\frac{1}{D}\int_{0}^{t}\int_{\Omega \times \Omega }\left(\;dh\left(z,s\right)K_{l}^{-1}\left(z-z^{\prime }\right)\left[\nu \partial_{z^{\prime }}^{2}h\left(z^{\prime },s\right)+\frac{\lambda }{2}{\left[\partial_{z^{\prime }}h\left(z^{\prime },s\right)\right]}^{2}\right]\right)\;d^{d}z^{\prime }\;d^{d}z,$$

where the stochastic integral in (15) is to be understood in the Stratonovich sense, i.e., the following:

(16) $$\Delta S_{t}^{m}\left[h\right]=\lim_{N\to \infty }\Delta S_{N,t}^{m}\left[h\right],$$

where

(17) $$\Delta S_{N,t}^{m}\left[h\right]=\sum_{k=0}^{N-1}\int_{\Omega \times \Omega }\;\left(\left[h\left(z,s_{k+1}\right)-h\left(z,s_{k}\right)\right]K_{l}^{-1}\left(z-z^{\prime }\right)\left[\nu \partial_{z^{\prime }}^{2}h\left(z^{\prime },\bar{s}\right)+\frac{\lambda }{2}{\left[\partial_{z^{\prime }}h\left(z^{\prime },\bar{s}\right)\right]}^{2}\right]\right)\;d^{d}z^{\prime }\;d^{d}z,$$

with $\bar{s}=\left(s_{k+1}+s_{k}\right)/2$, and $s_{k}=kt/N$, k∈{0,1,…,N}. It is now clear that with such Stratonovich interpretation of (15), one has (compare the result with Equation (14))

(18) $$dh\left(x,t\right)=D\left[\int_{\Omega }K_{l}\left(x-x^{\prime }\right)\frac{\delta \Delta S_{t}^{m}\left[h\right]}{\delta h(x^{\prime },t)}\;d^{d}x^{\prime }\right]dt+\sqrt{2D}\;dB\left(x,dt\right).$$

In particular, considering formally the case of a spatially uncorrelated noise, $K_{l\to 0}\left(x\right)=\delta \left(x\right)$, one has

(19) $$dh\left(x,t\right)=D\frac{\delta \Delta S_{t}^{m}\left[h\right]}{\delta h(x,t)}\;dt+\sqrt{2D}\;dB\left(x,dt\right),$$

which clearly shows that if the potential Φ is understood as a Stratonovich integral, then $\Phi \left[h\right]=D\Delta S_{t}^{m}\left[h\right]$. Also notice that only within the Stratonovich interpretation, one has

(20) $$\int_{0}^{t}\int_{\Omega }\;dh\left(z,s\right)\nu \partial_{z}^{2}h\left(z,s\right)d^{d}z=\frac{\nu }{2}\int_{\Omega }\left({\left[\partial_{z}h\left(z,t\right)\right]}^{2}-{\left[\partial_{z}h\left(z,0\right)\right]}^{2}\right).$$

As a plot twist, Equation (15) illuminates the “correct” way to define the potential, in the case in which the noise is correlated, for the thermodynamic interpretation to hold.

### 4.2. Memory of Initial Conditions in KPZ

The dependence of the asymptotic *h*-statistics on initial conditions is an established fact [30,31,32,33,44]. A numerical test, undertaken with initial conditions of the form

$$\begin{array}{ccc} h(0) & = & \frac{1}{a}\;\mathrm{abs}\left(x-L/2\right),\\ a & \in & \{0.5,1.5,3,20\}. \end{array}$$

shows that the NEP also displays memory effects.

**Figure 9:**
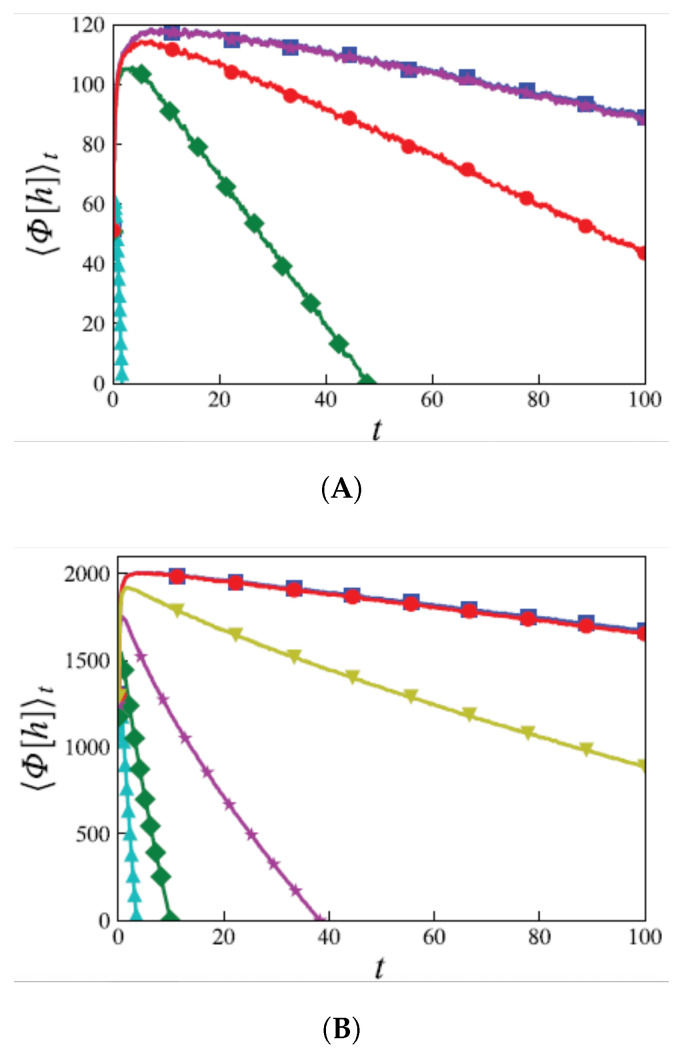
Ensemble average of Φ[h] in Equation (13), for λ=0.1. (**A**): a=0.5 (light blue), 1.5 (dark green), 3 (red), 20 (dark blue), and ∞ (magenta). (**B**) a=0.75 (light blue), 1.0 (green), 1.5 (magenta)), 3 (light green), 20 (dark blue), and ∞ (red). Figure (**A**) corresponds to d=1 while (**B**) corresponds to d=2.

Within the studied interval, the slopes show a strong dependence on the initial conditions, and in some cases, the curves also display concavity (Figure 9).

### 4.3. The Related Golubović–Bruinsma Model

As indicated in the Introduction, Golubović and Bruisnma [16] have analyzed an extension of the KPZ model. The equation for the the growth rate they have found is as follows:

(21) $$\frac{\partial h(x,t)}{\partial t}=\nu \nabla^{2}h\left(x,t\right)-{\left(K\nabla^{2}\right)}^{2}h\left(x,t\right)+\frac{\lambda }{2}{\left(\nabla h(x,t)\right)}^{2}+\xi \left(x,t\right).$$

As in the KPZ case, this equation can be derived from a NEP, given by

(22) $$\Phi_{GB}\left[h\right]:=\frac{\nu }{2}\int dx{\left(\nabla h\right)}^{2}+\frac{K}{2}\int dx{\left(\nabla^{2}h\right)}^{2}-\frac{\lambda }{2}\int dx\int_{0}^{t}ds\;\partial_{s}h{\left(\nabla h\right)}^{2},$$

such that

(23) $$\frac{\partial h(x,t)}{\partial t}=-\frac{\delta \Phi_{GB}\left[h\right]}{\delta h(x,t)}+\xi \left(x,t\right).$$

Equation (23) expresses the Golubovic–Bruinsma model as a stochastic gradient flow driven by the functional derivative of the non-equilibrium potential $\Phi_{GB}\left[h\right]$. This formulation highlights the shared variational structure between this model and the standard KPZ equation, allowing for a unified analysis of their dynamical and statistical properties within the same theoretical framework.

From this form of the potential, we can immediately predict that, for plane initial conditions, the long time behavior will be dominated by a linear dependence on *t*, with a slope depending on $\lambda^{2}$. We can also anticipate the existence of memory effects.

In the following figures, we show the time behavior of the indicated functional as well as, changing initial conditions, we prove the existence of memory effects. In all these figures, the parameters we have used are ν=λ=K=1.

**Figure 10:**
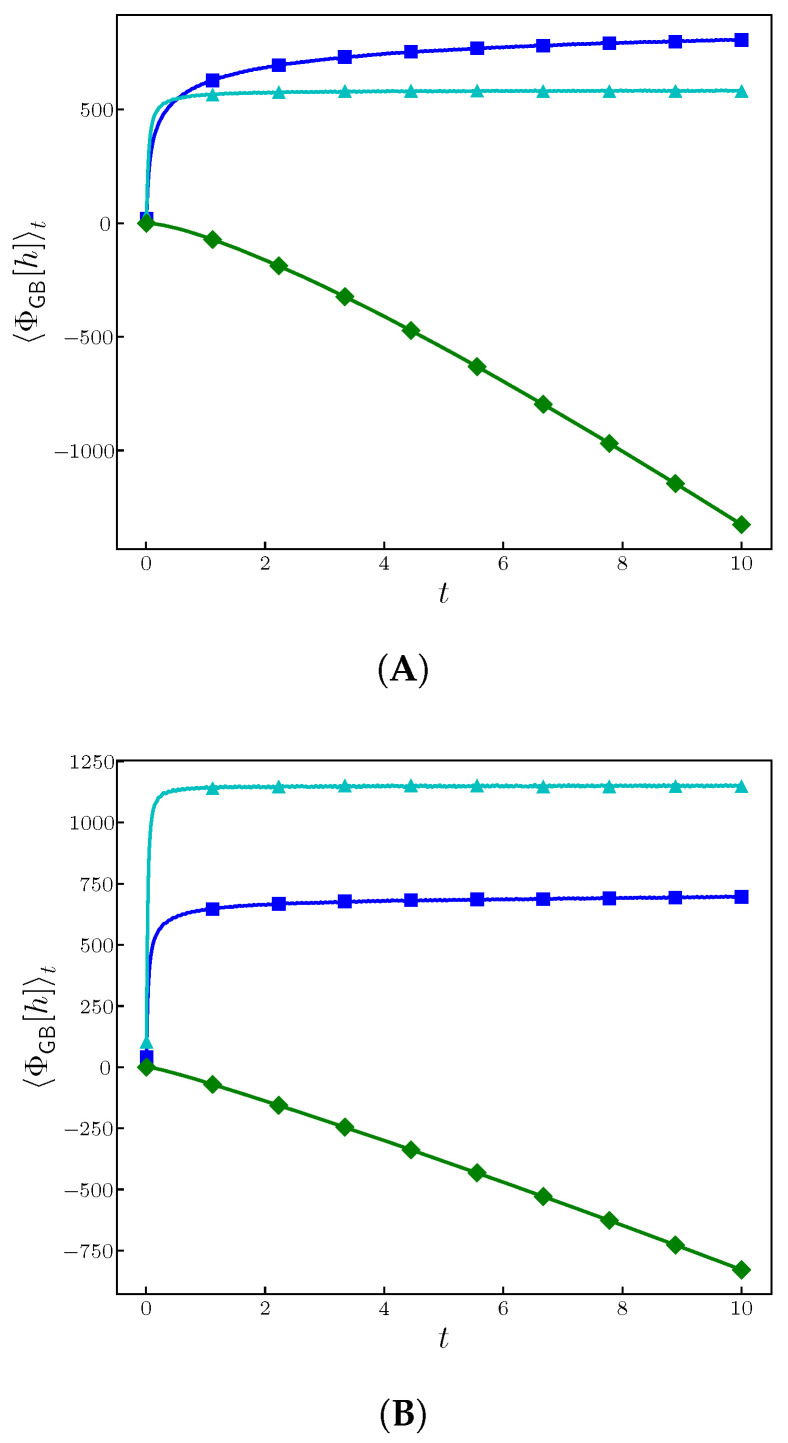
Ensemble average of $\Phi_{GB}\left[h\right]$ given in Equation (22) for ν=λ=K=1. On top we show the ((**A**), d=1) results, while on the bottom, the ((**B**), d=2) ones. The sizes used were L=4096 in (**A**) and L×L=64×64 in (**B**). We have shown the different components: blue and sky blue correspond to the linear contributions, while the green corresponds to the nonlinear one.

From Figure 10, it is clear that the linear contributions have a similar behavior than in the EW case, while the nonlinear contribution, dominating at long time, reaches a linear dependence on *t*. The slope should scale with $\lambda^{2}$, which is an aspect to be discussed in a forthcoming work.

**Figure 11:**
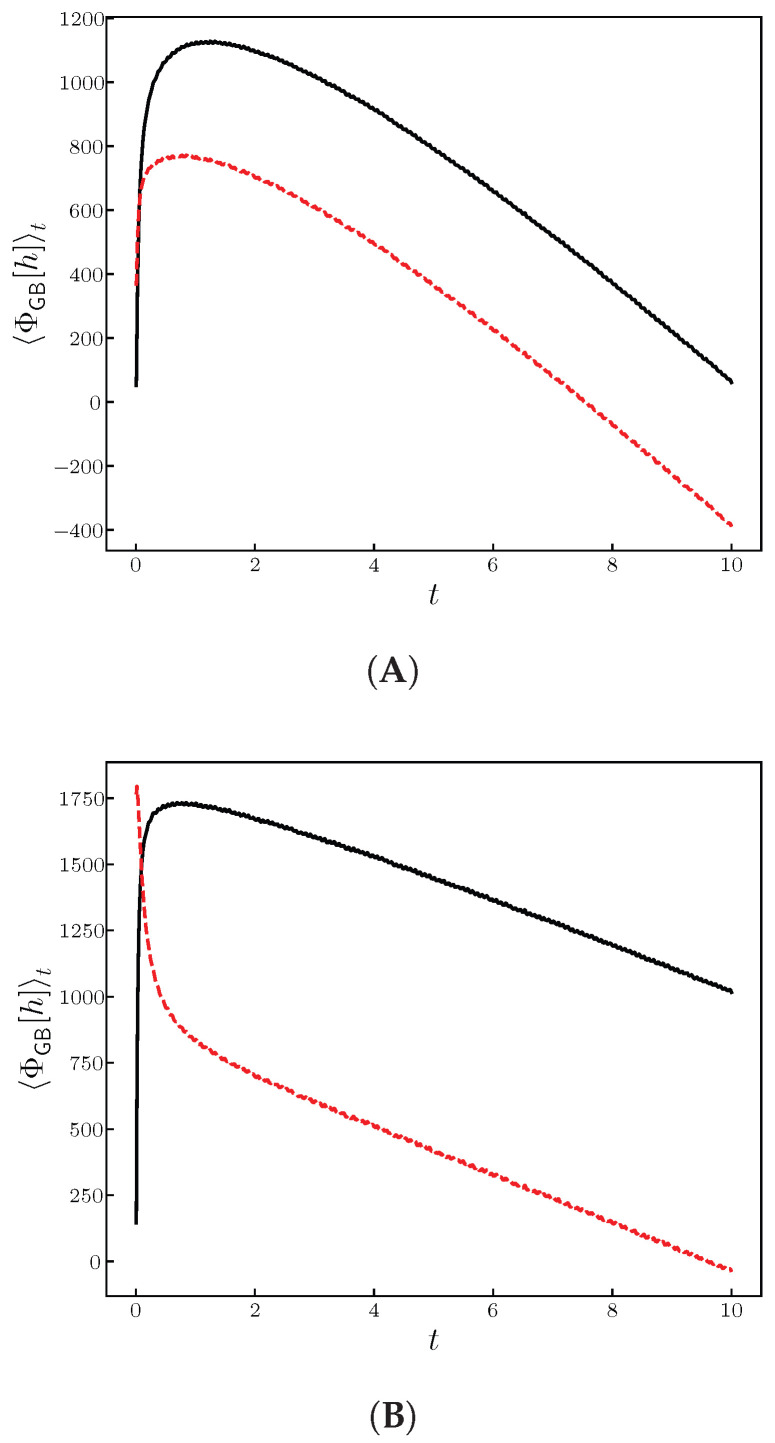
Ensemble average of $\Phi_{GB}\left[h\right]$ given in Equation (22) for different initial conditions. On top we show the ((**A**), d=1) results, while on the bottom, the ((**B**), d=2) ones. The sizes and parameters used were the same as in the previous figure. We have used flat i.c. indicated in black, and wedge ones (all sites =0, except the center that has the value 1) indicated in red.

In Figure 11, the memory effects are clearly shown when changing the initial conditions. A detailed study will be the subject of a forthcoming work.

Considering the associated Fokker–Planck equation, and considering the stationary case we observe that, independently of the bc, the exponential

$$P_{BG}^{\mathrm{stat}}\left[h\right]\propto \exp\left\{-\Phi_{KPZ}\left[h\right]/\varepsilon \right\},$$

fullfils the stationary condition

$$\frac{\partial }{\partial t}P_{BG}^{\mathrm{stat}}\left[h\right]=0,$$

with $\Phi_{BG}^{\mathrm{stat}}\left[h\right]$ given by Equation (22). Clearly, as in the KPZ case, this is a *formal* solution since $P_{BG}^{\mathrm{stat}}\left[h\right]$ *cannot* be normalized.

An interesting aspect was studied in [45] and corresponds to the *large deviations of the interface height* in this model. Such a study follows [46,47] on similar aspects for the KPZ equation.

### 4.4. Isolating the Collective Mode

This prima facie promising approach was also worked out in [40,41]. By writing $h\left(x,t\right)=\bar{h}\left(t\right)+y\left(x,t\right)$, with

$$\bar{h}\left(t\right)=V{\left(\Omega \right)}^{-1}\int_{\Omega }dx\;h\left(x,t\right),$$

and being $\nabla \bar{h}=0$, Equation (2) becomes

(24) $$\partial_{t}y\left(x,t\right)=\nu \;\nabla^{2}y\left(x,t\right)+\frac{\lambda }{2}\;{\left[\nabla y\left(x,t\right)\right]}^{2}+F+\xi \left(x,t\right)-\dot{\bar{h}}.$$

Equation (2) yields $\dot{\bar{h}}=F+G\left(t\right)$, with

$$G\left(t\right):=\frac{\lambda }{2V(\Omega )}\int_{\Omega }dx\;{\left[\nabla h\left(x,t\right)\right]}^{2}+\bar{\xi }$$

parametrically depending on λ and *F*. Note the term $\bar{\xi }$, whose variance is depressed from ϵ by V(Ω). Hence Equation (24) can be written in the form

(25) $$\partial_{t}y\left(x,t\right)=\nu \;\nabla^{2}y\left(x,t\right)+\frac{\lambda }{2}\;{\left[\nabla y\left(x,t\right)\right]}^{2}-G\left(t\right)+\xi \left(x,t\right).$$

Unfortunately, the inherent constraint $\int_{\Omega }dx\;y\left(x,t\right)=0$ hinders the usefulness of this otherwise promising approach.

## 5. Conclusions and Outlook

Here we highlighted, from a variational approach, several aspects of the KPZ equation as a theory of rough interface growth. We have addressed several misconceptios about KPZ—such as the alleged absence of a free-energy-type functional (the NEP), the supposed role of Galilean invariance in fixing the universality class, the Markovian character of the process, and the conjecture linking the stationary EW pdf to d=1 KPZ with periodic boundaries—and dismantled them.

In addition, we presented numerical results of the NEP (from which one can extract information on the λ dependence of its long-time behavior and the crossover time from EW to KPZ regime), and have shown numerically that also the NEP displays memory of initial conditions (a fact established in several theoretical works). We have also referred to our work on the stochastic thermodynamic description of KPZ based on a variational approach, allowing us to numerically obtain several known fluctuation theorems and TUR for entropy production.

We have also shown the existence of a NEP in a related model, the Golubovic–Bruinsma one. We have shown the time dependence of such a functional, as well as the existence of memory effects. We expect to extend this approach to other related systems. We also expect to exploit our functional approach to investigate large deviation functions as discussed in [45,46,47].

We are currently working on the possibility of obtaining information about the critical exponents and the existence of an upper critical dimension, using the time behavior of the NEP. While RG fails for d>1, the variational approach offers a qualitative or complementary insight, even if exact exponents are still an open challenge.

## Data Availability

No new data were created or analyzed in this study.
